# The human gut microbiome and aging

**DOI:** 10.1080/19490976.2024.2359677

**Published:** 2024-06-03

**Authors:** Evan Bradley, John Haran

**Affiliations:** UMass Chan Medical School, Department of Emergency Medicine and Department of Microbiology and Physiologic Systems, Program in Microbiome Dynamics, Worcester, MA, USA

**Keywords:** Gut microbiome, aging, age-related disease, microbiome-based theraputics

## Abstract

The composition of the human gut microbiome has been observed to change over the course of an individual’s life. From birth, it is shaped by mode of delivery, diet, environmental exposures, geographic location, exposures to medications, and by aging itself. Here, we present a narrative review of the gut microbiome across the lifespan with a focus on its impacts on aging and age-related diseases in humans. We will describe how it is shaped, and features of the gut microbiome that have been associated with diseases at different phases of life and how this can adversely affect healthy aging. Across the lifespan, and especially in old age, a diverse microbiome that includes organisms suspected to produce anti-inflammatory metabolites such as short-chain fatty acids, has been reported to be associated with healthy aging. These findings have been remarkably consistent across geographic regions of the world suggesting that they could be universal features of healthy aging across all cultures and genetic backgrounds. Exactly how these features of the microbiome affect biologic processes associated with aging thus promoting healthy aging will be crucial to targeting the gut microbiome for interventions that will support health and longevity.

## Introduction

The gastrointestinal microbiome is the collection of some 10^1^ bacterial cells that reside within the human gastrointestinal tract representing more cells that are contained within the human body, and a metagenome (combined genome of these commensal organisms) that is far larger than the human genome.^2^ This community of organisms has been observed to change over the lifespan ,^3 ,^ .^4,5^ Machine-learning-based analysis of published gut microbiome datasets could predict a subject’s chronologic age within 5.9 years, although this study was conducted largely independent of any health information.^6^ A cross-sectional study across the lifespan (age 1 to over 100) in a Japanese population showed a characteristic microbiome within infants and young children prior to weaning, then a transition to a more diverse microbiome associated with introduction of solid foods. This diverse and dynamic microbiome develops until early adulthood and then becomes relatively stable when it begins to show a decline in diversity after peaking late in life (around 65) and becoming more pronounced in individuals older than 80 years.^4^ An Italian study of individuals across the mirrored many of these findings including a unique microbiome among young children with characteristic taxa and relatively low diversity, and then a transition to a more diverse, adult microbiome in adolescents. They also observed microbiome signatures that became more unique to the individual at extremes of age^7^ which may reflect the microbiome becoming tailored to the individual’s diet and living environment that perhaps varies less at extremes of age. Intriguingly, very long-lived individuals (over 100 years old) have shown a distinct gut microbiome profile with greater diversity a high abundance of health-associated taxa such as *Christensenellaceae* and *Akkermansia*^*7*^. Although the gut microbiome changes across the lifespan, there are features that have been associated with diseases that develop at different phases of life and contribute to the development of age-related disease later in life. Of particular interest are the microbiomes of “super-agers” who reach extremes of age in relatively good health and have the potential to offer insights into how the microbiome can affect longevity and resistance to age-related diseases. Some summaries of the findings of microbiome features associated with very old adults are summarized in Table 1.

**Table 1.** Summary of studies of microbiome features associated with healthy aging in very old cohorts.

Study Subjects	Microbiome Findings	Reference
Older adults 70–82 examining cohorts with and without underlying chronic disease	Higher abundance of *Akkermansia muciniphila* by 16s rRNA sequencing	Singh et al. (2019)
A subset of much larger cohorts comprising individuals 85 year and older living in the community	Unique microbiomes enriched in rare taxa by 16s rRNA sequencing and increased serum concentrations of microbial metabolites of aromatic amino acids	Wilmanski et al. (2021)
Comparison between older adults and centenarians	Increased abundance of metabolic pathways associated with tryptophan metabolism by metagenomic sequencing	Rampelli et al. (2013)
Comparisons between young adults and cohorts up to semisupercentenarians	Increased metabolic pathways associated with xenobiotic degradation, decreased pathways associated with carbohydrate metabolism by metagenomic sequencing in older cohorts	Rampelli et al. (2020)
Comparison between centenarians and young adults living in rural India	Higher bacterial richness and increase in *Prevotella* strains by 16s rRNA sequencing and higher concentration of neurotransmitter related metabolites in stool	Tuikhar et al. (2019)
Comparisons between young adults and cohorts up to semisupercentenarians	Increased abundance of *Christensenellaceae* and *Akkermansia* in the oldest cohort by 16s rRNA sequencing	Biagi et al. (2016)

## The microbiome in infancy and childhood and impacts on health later in life

The development of the gut microbiome may begin before birth as the fetus is likely exposed to microbial metabolites from the mother, if not actual bacterial communities, *in utero* .^8^ At birth, there is a major transfer of microbiota from mother to baby that is greatly affected by the mode of delivery (vaginal vs. cesarean section).^9^ The transfer of microorganisms continues and is affected by whether the baby is breast or formula fed^10^ with bacterial species possibly being directly transmitted through breast milk.^11^ In a large international longitudinal study of young children, breast-fed infants show a distinct microbiome compared to formula-fed infants, with higher abundances of *Bifidobacterium* and pathways associated with fatty acid biosynthesis.^12^ The next major transition in the microbiome occurs as solid food is introduced and the child is weaned from breast milk or formula.^12,13^ The microbiome is now influenced by diet and the environment to which the child is exposed. For example, exposure to household disinfectants early in life was associated with a higher abundance of *Lachnospiraceae* and subsequently the development of higher body mass index in a study of a Canadian cohort.^1^ The make-up is also affected by other variables in the environment, such as siblings and pets.^12^

The composition and acquisition of the microbiome early in life is of great interest because it is thought to affect the development of diseases later in life.^14^ The microbiome may be affected by prenatal exposures^15^ and starting at birth, vaginal delivery versus cesarean section (c-section) has large impacts on early microbiome. Children born by c-section show early acquisition of more skin flora and facultative anaerobes and have delayed acquisition of obligate anaerobic organisms. These obligate anaerobes produce microbial products typically associated with a healthy microbiome like short-chain fatty acids (SCFAs).^16^ SCFAs are produced through fermentation of non-digestible carbohydrates and are thought to be an important microbial metabolite that may have beneficial effects across the lifespan.^5^ SCFAs are thought to shift immune cells toward an anti-inflammatory profile^17^ and have been implicated as protecting against a wide range of diseases.^18^ Although many c-section delivered infants “catch up” in terms of acquiring these organisms, there may be changes in the composition of anaerobic organisms that persist even into adulthood.^19^ Some taxa within the family Bacteroidaceae, shown to vary between c-section and vaginal delivery group, were also associated with reduced head-circumference growth, an early marker of cognitive development, that also likely has long lasting consequences into adulthood.^20^

Differences in the gut microbiome taxa have been noted in infants and young children who develop life-long diseases that include eczema,^21^ asthma,^22^ and type 1 diabetes.^23^ Many of these childhood diseases are related to a disordered immune response, and the microbiome is thought to play a role in early immune system development that could prevent the development of autoimmune diseases as well as inflammatory bowel diseases later in life.^14^ Indeed, the presence of SCFA producing organisms appeared to be protective when comparing children who developed type 1 diabetes to those who did not.^24^ The abundance of genes for the production of a lipopolysaccharide (LPS), a highly inflammatory component of the outer membrane of Gram negative bacteria,^25^ and the concentration of microbial products such as secondary bile acids and amino acids were higher in the gut microbiomes of infants who went on to develop atopy and asthma.^22^

Exposures that affect the microbiome, such as antibiotics or the lack of a varied and healthy diet, unquestionably impact the microbiome of children and can be seen in reduced diversity and increased pathobionts, organisms that contribute to maladaptive process such as stunted growth and inflammation.^26,27^ This can impact development of disease later in life as well. This was illustrated by a study of a cohort of children from the UK that examined risk of development of inflammatory bowel disease. This analysis showed that there was an increased risk of inflammatory bowel disease in those with exposure to antibiotics active against anaerobes and that the risk was greater if the child was treated at a younger age.^28^ A study of a separate cohort from the USA showed antibiotic exposure within the first 2 years of life was associated with a wide range of diseases including allergic/inflammatory conditions like asthma, atopic dermatitis, metabolic disease including obesity, and even attention deficit hyperactivity disorder.^29^

**Figure 1.**
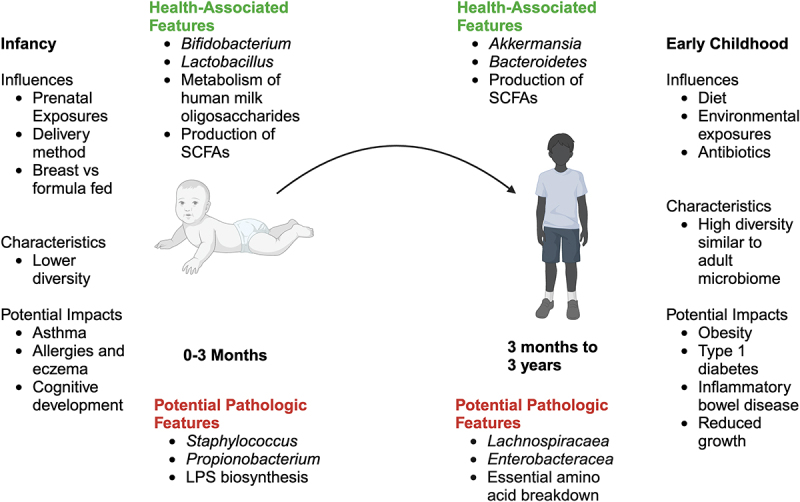
Influences, characteristics, and potential health impacts of the gut microbiome among infants and children.

A summary of characteristics, influences, health associated features and potential pathologic features of the gut microbiome among infants and young children is shown in Figure 1.

## Adult microbiome

The adult microbiome appears to be highly personalized and stable and does not drastically change with short-term variations in diet.^30^ In general, among adults, a diverse gut microbiome that contains a large number of species (high diversity) as well as high abundance of numerous species (high evenness) is healthy and robust one.^31,32^ Microbiome diversity may signal more than just the absence of disease as elite athletes have been shown to have more diverse microbiomes.^33^

Although the established adult microbiome is observed to be fairly stable, external factors, likely primarily diet,^34^ affect the composition of the adult microbiome over the longer term. This is well illustrated by the study of adult immigrants to the USA from Asia which compared microbiome profiles of immigrants, and the children of immigrants, to individuals from the same ethnic group in their country of origin.^35^ This study showed a shift in the microbiome that correlated with the duration of time the individual had been a resident of the USA, including a loss of overall diversity and loss of abundance of *Prevotella* strains associated with metabolizing fiber, and an increase in *Bacteroidetes*. The longer a subject had been a resident of the USA, the more similar their gut microbiome was contemporary US residents. In this study, diet of study subjects was significantly, although not entirely, associated with the shift toward contemporary USresidents, suggesting that diet was an important component of this change, but not solely responsible. Dietary patterns have also been associated with increased abundance of health associated taxa and a decrease in potential pathobionts. An 8-week intensive dietary intervention based on the Mediterranean Diet^36^ leads to increases in abundance of health associated taxa like *Faecalibacterium prausnitzii*, an SCFA producing organism frequently cited as being associated with positive health markers,^37^ and a decrease in *Rumminococcus gnavus*, a potential inducer of gut inflammation.^38^

Physical activity can also alter the microbiome at both a taxonomic level and with respect to microbial metabolites.^39^ Adults with an active lifestyle tend to show higher diversity and abundances of health-associated taxa than sedentary adults.^40^ The microbiomes of professional athletes also showed an increased abundance of metabolic pathways associated with SCFA production compared to sedentary controls.^41^ The gut microbiome may also be able to determine how an individual responds to a physical activity-based intervention, in a study of 39 individuals with pre-diabetes, response to the intervention in terms of fasting blood glucose was associated shifts in microbial taxa and metabolic pathways, while those who did not respond to the intervention resembled sedentary controls.^42^ It is worth noting that interventional studies examining the effect of exercise on the microbiome have shown that positive changes are dependent on ongoing activity and are lost once activity is ceased.^39^ In older adults specifically, physical activity-based interventions can cause an increase in health-related taxa but do not impact broad diversity measures.^43^

During adulthood, specific taxa and overall microbiome diversity are associated with markers of good health and protection against the development of chronic health conditions. An adult’s weight is also correlated with the development of many chronic conditions, and microbiome features have been identified that correlate with obesity.^44^ A higher *Firimicutes* to *Bacterodetes* ratio has been consistently reported as being associated with obesity.^44^ A higher microbiome diversity has also been associated with less weight gain over the lifespan.^45^ Among adults, the genus *Christensenellaceae* has been correlated with good metabolic health including lower body mass index,^46^ healthy serum lipid profiles,^47^ and reduced adipose tissue.^48^ The genus *Prevotella* is thought to be involved in metabolizing fiber, often lacking in Western diets, and determine if an individual will respond favorably to a dietary intervention increasing their intake of fiber.^49^ *F. prausnitzii* was also found to be higher in abundance in lean individuals.^50^ Correlations between the composition of the gut microbiome and numerous conditions that develop during this phase of life have been reported on including, cardiovascular disease,^51,52^ kidney disease,^53^ and type 2 diabetes.^54^ The risk for these disease increases with age, and they contribute to the development of age-related diseases later in life.^55^ Age-related disease are those that occur later in life such as dementia and malignancies and are thought to be related to, although not necessarily dependent on, the biological aging process.^56^ Specific dementia syndromes like Alzheimer’s disease are also associated co-morbidities that develop in this phase of life such as cardiovascular disease.^57^

## The older adult microbiome and healthy aging

**Figure 2.**
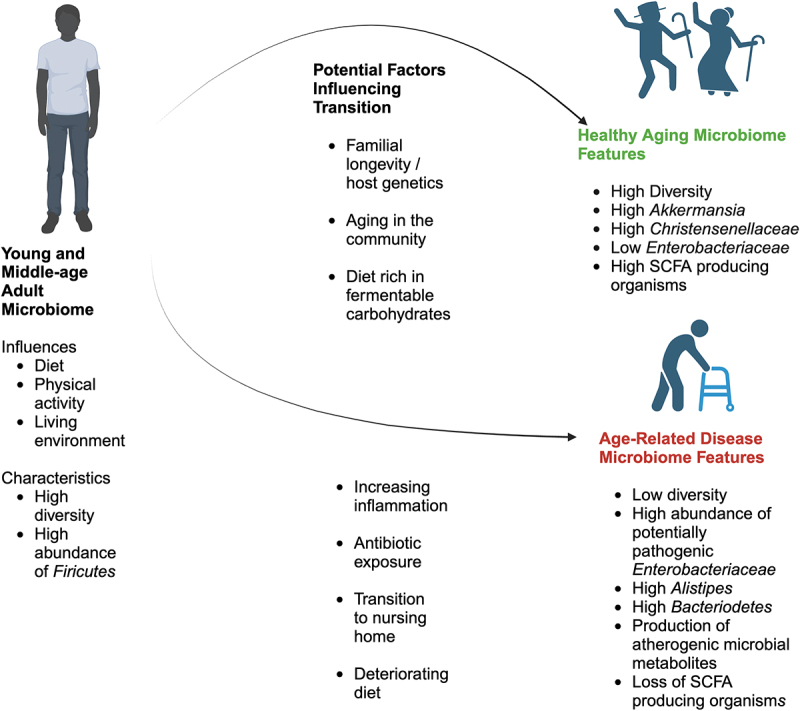
Influences and characteristics of the adult microbiome and factors influencing transition to a healthy or age-related disease older adult microbiome.

Compared to younger individuals, studies conducted on older adults in the USA and Europe tend to have lower overall taxonomic diversity within their microbiomes and have higher abundances of *Bacteroidetes*.^3,58,59^ When very old adults’ (90 years of age and older) microbiomes are examined, the make-up of the microbiome is likely highly dependent on the health status of the individual, with healthy-longer lived individuals showing unique microbiome signatures.^60^ This suggests an age-related deterioration of the microbiome similar to the age-related decline of other biologic process. This general age-related shift in the microbiome is distinct from co-incident health conditions.^61^ It has been suggested that there are microbial taxa associated with healthy aging, and that loss of these may represent “taxonomic milestones” that contribute to or accompany physiologic decline and subsequent age-related disease.^62^ A summary of the potential exposures that contribute to a gut microbiome that reflects healthy aging as opposed to one that reflects age-related disease and salient microbiome features of these two states is shown in Figure 2.

Although age itself likely contributes to changes in the gastrointestinal microbiome, it is also greatly impacted by the environment in which an individual lives and ages. An exploration of data from metagenomic sequencing of microbiome samples across Europe, Africa, North and South America showed that there were distinct features of each geographic area throughout the lifespan.^7^ Much of the literature notes changes in taxa with age that tends to be conserved across different areas of the world. There are, however, notable differences depending on the region in which the study was undertaken. Studies of cohorts in Italy^63^ and Ireland^64^ have shown decreased abundances of *Roseburia* with aging, while studies cohorts in Korea^65^ and China^66^ have reported increases in this genus. *Bacteroidetes* are generally described as increasing with age, but the converse was observed among healthy older Indonesians.^67^ A study of healthy centenarians from India and comparing them with studies previously mentioned from Italy, China, and Japan found unique features in the Indian population such as lower Bacteroidetes, higher *Enterobacteriaceae* among the Indian cohort.^68^ *Akkermansia*, usually associated with healthy aging, was associated with frailty in a cohort of Chinese older adults.^69^ These discrepancies highlight the extremely complex relationship between the gut microbiome and aging, which is affected not only by the myriad interactions between the host-specific organisms in the microbiome, but also the diet and environment in which the individual ages. In an attempt to examine relationships between aging across published datasets, Ghosh et al. examined 21,041 fecal microbiomes across seven different datasets representing Europe, North and South America, and Asia.^70^ They identified a higher Kendal Uniqueness score, which is characterized by a loss of typically dominant microbiome taxa and an increase in subdominant taxa, as associated with unhealthy aging. They also identified taxa that were associated with a higher Uniqueness score, including members of *Klebsiella, Ruminococcus*, and *Clostridium* genera were generally those reported as being associated with dysbiosis and an unhealthy gut microbiome. Taxa that were negatively associated with the Uniqueness score, including members of *Corprococcus*, *Akkermansia* and *Faecalibacterium*, were generally those that are associated with good health and healthy aging. This highlights that there are recurring microbiome trends across geography and cultures that are associated with healthy aging.

Diet and the living environment of an individual can cause shifts in the microbiome.^34,35,71^ The environment that older adults occupy in society varies across region and socio-economic status. In lower and middle income countries, older adult care is often managed by family units within the community,^72^ although this is changing with increasing economic development.^73^ In the developed world, a significant portion of older adults will live at least some part of their life within a long-term residential medical facility or nursing home.^74^ In the USA, the longer an individual lives, the greater chance they have of spending part of their life in a nursing home.^75^ This context is important to consider when examining the results of microbiome analysis from older adults in different parts of the world.

Insights into what causes shifts away from health-associated taxa and diversity as an individual ages have come from examining the microbiome of older adults shifts when they enter a nursing home, typically associated with a decline in health. Lower microbiome diversity and an increase in the abundance of potential pathogens is seen in older adults who reside in nursing homes^3,64^ These individuals also tend to have more chronic health conditions compared to community-dwelling older adults.^76^ The observed shift from a community-dwelling adult’s microbiome to a microbiome that resembles a resident of a long-term facility occurs over the course of a year residing in a facility.^58^ Nursing home residents followed over time also show gradual decreases in commensals associated with SCFA production such as *F. prausnitzii* and increases in pathobionts like *Veilonella parvula* .^77^ These shifts are to be likely partially attributable to dietary changes that occur when an individual enters long-term care where diets are typically lower in fiber.^64^ Older adults who reside in nursing homes are very frequently hospitalized and treated with antibiotics.^78^ Antibiotics can have a profound and long-lasting effect on the microbiome, notably a reduction in microbial diversity.^79^ As a potential direct consequence of this, the microbiome can come to harbor an increased abundance of pathobionts and antibiotic-resistant pathogens that can later cause difficult to treat infections.^80,81^ This may also lead to a microbiome that is more favorable to pathogen colonization, such as *Clostridiodies difficile* colonization.^82^ Older adults who are colonized or suffering from *C. difficile* colitis show increases in pathobionts and decreases in beneficial commensals that share some similarities with changes described more generally with aging.^83,84^

The degradation or “unhealthy aging” of the microbiome may not be an inevitable process, as illustrated by studying individuals who stay healthy as they age. A large cross-sectional cohort in China showed that healthy young adults and healthy older adults had very similar microbiome profiles even when extended to ages beyond 94 years.^85^ In some studies, healthy long-lived individuals have a higher gut microbiome diversity even than younger individuals^86,87^ suggesting that a healthy microbiome can contribute to resistance to age-related disease, and hence, longevity. With regard to specific taxa, a higher abundance of *Akkermanisa*, generally reported to decrease as individuals age,^88,89^ has been associated healthy aging.^90^ *Christensenellaceae* may be another genus that contributes to healthy aging and longevity, although it generally declines as an individual ages, but has been observed in high abundance in very long-lived individuals (greater than 100 years of age).^63^ Among very long-lived individuals (centenarians and super centenarians) studies across geographic regions have documented high diversity and abundance of *Lachnospiraceae*, *Ruminococcaceae*, and *Akkermansia* ,^91^ all thought to be beneficial taxa. A high species richness (number of bacterial species represented), especially among the family *Ruminococcaceae*, may also be conserved feature across the long-lived cohorts.^68^ A 2018 cohort of cognitively healthy centenarians recruited in the Netherlands included a study of the microbiome of this cohort, the results are yet to be published at the time of this review, but could hold important insights into the features of these so-called “super-agers”.^92^ There have also been associations between microbiome features and specific age-related diseases. A*listipes*, a genus whose abundance has been described as increasing with age,^93^ has been associated with dementia.^94^

With increasing age, older adults can develop decreased resilience to stressors like acute illness or injury, which is conceptualized as frailty.^56^ Frailty is a multi-domain functional status which includes chronic disease burden as one dimension related to global wellbeing and function of the individual.^95^ Frailty is typically measured as an ordinal score by geriatric health care providers with scores meant to describe anywhere from a very fit individual to one who is terminally ill.^96^ A longitudinal study of older adults has shown a decrease in *F. prausnitzii* and increases *Eggerthella* and *Eubacteria*, associated with increasing frailty.^97^ An examination of the gut microbiome of residents from a nursing home found decreased *Lachnospiracea*, usually considered a beneficial taxa, and increased abundance of *R. gnavus*,^89^ previously mentioned as proinflammatory. A study of community dwelling older adults in South Korea found non-significant decreases in alpha diversity and decreases in species of *Prevotella copri*, a genus associated with fermentation of fiber, and *Corprococcus eutactus*, a potential SCFA producing organisms, were associated with increasing frailty.^98^

A finding that has been quite consistent among the studies of age-related disease is decreased microbiome diversity,^89,97^ but it is important to point out that associations with certain taxa are not always consistent across studies. *Corprococcus* has generally been associated with healthy aging^70,99^ but was also decreased in a cohort that stayed healthy into very advanced age.^100^ *Ruminococcus* is a genus that contains members that produce SCFAs and are thought to be anti-inflammatory^101^ were seen to be increased in abundance in centenarians,^68^ but also has members associated with frailty.^89,102^ *Lachnospiracea*, usually reported as being associated with healthy aging, was associated increased BMI in children.^1^ This likely reflects two issues with research into the gut microbiome among humans: 1) its observational nature and 2) a general lack of understanding of mechanistic insights into how the microbiome impacts specific health outcomes. Because majority of research on aging and the gut microbiome is observational it is impossible to know if unseen variables are confounding the association between specific taxa and positive outcomes. Microbiome research is making progress into the mechanisms by which the gut microbiome impacts specific process, which we discuss in the next section. Understanding these mechanisms will be crucial to harnessing the microbiome for treatment.

## Gut microbiome and immune system implications

An important consideration is how changes or “degradation” of an aging microbiome may impact biologic processes that are associated with aging. Many characteristic age-related diseases are thought to be related to chronic low levels of inflammation; a hypothesis known as “inflammaging”.^55^ Inflammation has been shown to be associated with a wide variety of diseases whose risk increases with age such as cardiovascular disease,^103^ metabolic syndrome,^104^ and chronic kidney disease.^105^ The inflammation associated with aging also impacts physiologic process, such as muscle protein synthesis and insulin sensitivity, that can lead to sarcopenia and contribute to frailty.^106^ The burden of these diseases accumulates contributes to the development of dementia and frailty as an individual ages.

Inflammation associated with aging likely drives changes the gut environment to some extent and drives changes in the microbiome.^91^ Higher levels of inflammation have been associated with specific taxa in the microbiome such as potentially pathogenic organisms belonging to *Escherichia* and *Klebsiella* among older adults.^107^ Similar species are prominent in the highly-inflamed environment of the gut in sufferers of Inflammatory Bowel Disease.^108^ These taxa are potential pathogens and have strategies to survive immune effectors^109^ that may be present in a highly inflamed environment, may also promote inflammation leading to positive feedback that reinforces this environment.^110^ There are also taxa that are thought to be anti-inflammatory. Originally discovered in Crohn’s disease patients as being associated with a decreased risk of disease recurrence, *F. prausnitzii* has demonstrated strong anti-inflammatory effects *in vivo* .^111^ This species is also decreased in older adults with cognitive impairment due to the systemic pro-inflammatory environment in these patients.^112^ Studies of older adults and super-agers that have reached the extremes of aging have shown microbiome features that are associated with elevated levels of inflammatory cytokines,^69,107^ potentially anti-inflammatory bioactive metabolites,^113^ and decreased expression of genes important in limiting the inflammatory response to commensal organisms.^94,114^ Studies linking microbiome features with measures that impact systemic inflammation specifically among older adults are summarized in Table 2.

**Table 2.** Summary of studies examining interaction between the gut microbiome and inflammation in older adults.

Study Subjects	Microbiome Findings	Immune/Inflammatory Findings
Gong et al. 2023 30 long lived (greater than 90 years) 25 Old elderly (75–80) 75 Young Elderly (60–74) 15 Middle Age (Less than 59)	Decreased abundance of butyrate producing members of the *Clostrida* class, increased abundance of *Kleibsiella* was correlated with increasing age	Measurement of fecal metabolites across different groups showed an increased abundance of a thromboxane analogue among long lived individuals that stimulated phagocytosis and an anti-inflammatory phenotype in microglia *in vitro*
Haran et al. 2019 108 older adult residents of nursing homes	Increased abundance of *Bacteroides*, *Alistipes, Odoribacter, Barnesiella*, and decreased abundance of *Lachnoclostridium* among older adults with a diagnosis of dementia	Decreased expression of p-glycoprotein in intestinal epithelial cell cultures when treated with stool supernatants from subjects with a dementia diagnosis
Xu et al. 2021 94 community dwelling older adults residing in the community	Increased abundance of *Akkermansia*, *Parabacteroides*, and *Klebsiella* and lower levels of the *Faecalibacterium*, *Prevotella*, *Roseburia*, *Megamonas*, and *Blautia* among frail individuals	Increased serum levels of IL-6 was seen among frail adults and was specifically associated with the abundance of *Escherichia/Shigella*, *Pyramidobacter*, *Alistipes*, and *Akkermansia*
Biagi et al. 2010 21 centenarians 43 older adults (59 to 78 years) 20 young adults (25 to 40)	Increased abundance of Proteobacter including *Escherichia*, *Proteus*, and *Kleibsiella* with increasing age	Elevated levels of IL-6 and IL-8 associated with pathobionts and potentially pathogenic organisms

One mechanism by which the gut microbiome impacts systemic inflammation may be impacting gut permeability. Increased intestinal permeability, usually measured by examining concentration of tight junction proteins or microbial products in serum, is hypothesized to lead to increased translocation of microbial products leading to inflammation.^115^ A small study of older adults with dementia found higher biomarkers of intestinal permeability associated with a diagnosis of dementia.^116^ Higher biomarkers of intestinal permeability have also been shown to be associated with increasing frailty.^117^ A decrease in SCFA producing organisms is usually associated with increasing intestinal permeability. It is thought that SCFAs are an energy source for healthy colonocytes^118^ and stimulate the maintenance of intestinal barrier function^119^ and this may be one mechanism for how they decrease systemic inflammation.

A seemingly contradictory immune process to inflammaging, immunosenescence, in which the aging immune system shows a decreased ability to respond effectively to pathogens,^120^ may also be influenced by the gut microbiome. A study of a middle aged and older adults in China showed microbiome features such as richness and abundance of *Akkermansia* were positively associated with higher circulating levels of IgG and IgA.^121^

## The metabolic capacity of the gut microbiome and health aging

Although taxonomy is a useful metric to examine microbiome composition, the functional metabolic capacity and metabolome represents what the collective microbiome’s functional capacity is, and metabolites produced by or modified by the gut microbiome can either interact locally within the gut or circulate and effect distant biologic process.^122^ This functional microbiome also changes as individuals age, as measured by abundance of genes representing metabolic pathways.^123^ The microbial-derived metabolites produced by the gastrointestinal microbiome, acting either locally or systemically are likely to determine a large number of host-microbiome interactions.^124^ This can be measured through serum metabolites thought to be modified or produced by the gut microbiome^125^ or directly measuring metabolites in the stool.^113^ As previously mentioned, SCFAs are a major metabolite produced by microbiota are thought to be anti-innflammatory^17,126^ and thus may counteract inflammation and inflammaging in the older adult. SCFAs are primary products of microbial fermentation of fiber, as this is the indigestible carbohydrate that reaches the large intestine.^127^ Diet has long been described as contributing to inflammation, with diets rich in fiber, such as the Mediterranean diet described as decreasing inflammatory markers.^128^ Older adults in microbiome studies, especially those in long-term care facilities, tended to have lower fiber diets,^64^ suggesting that a loss of SCFA producing bacteria may be diet-related. It has been suggested that a loss of SCFA producing bacteria contributes to inflammation in older adults^123^ and many of the beneficial taxa previously mentioned including *Akkermansia* and *Christensenellaceae* are potential short-chain fatty acid producing organisms.^63,88,89^ A cross-sectional study in China suggested that it may not just be loss of SCFA production capacity, but that an aging microbiome also had increased abundance of pathways associated with SCFA degradation, and this was associated with an increased abundance of pathobionts.^129^

Microbial metabolism of compounds in the diet also likely impacts health- and age-related disease. Polyphenols, antioxidant compounds typically found in nuts, fruits, and chocolates, are extensively metabolized by the gut microbiomes into compounds that may have greater bioavailability or distinct effects.^130^ A dietary intervention to supplement polyphenols in the diet of older adults showed decreases in measures of gut permeability and levels of inflammatory cytokines and markers of intestinal permeability. The gut microbiome can metabolize choline and phosphatidylcholine, primarily from animal products in the diet, to produce trimethylamine and subsequently trimethylamine-*N*-oxide (TMAO) which promotes atherosclerotic vascular disease.^131^ A study of a cohort in Germany suggested that microbial pathways were significantly contributing to serum TMAO and that TMAO levels and microbial pathways associated with TMAO synthesis increased with age.^132^ TMAO serum concentrations have also been inversely correlated with scores on cognitive neuropsychiatric test scores in older adults.^133^ Microbial breakdown products of aromatic amino acids have also been associated with atherosclerotic disease.^122^ In a study of familial long-lived individuals in China, distinct signatures of fecal metabolites were noted in long-lived individuals (greater than 90).^113^ One metabolite in particular, an analogue of the prostaglandin thromboxane A2, was enriched in families that tended to have long-lived individuals and showed pro-phagocytotic and potentially anti-inflammatory effects on central-nervous system derived phagocytes *in vitro*.^113^ However, thromboxane A2 can also be a mediator of thrombotic events due to cardiovascular disease,^134^ so the overall benefit of this metabolite is unclear. Another study, performed in China, showed that older members of the cohort had an increased abundance of LPS biosynthetic pathways^129^ and increased LPS production within the gut could contribute to previously discussed inflammaging.

Intriguingly, the gut microbiome may also function to mitigate environmental exposures, when examining supercentenarians specifically, older adults showed increased abundance of metabolic pathways associated with xenobiotic chemical degradation, suggesting that the microbiome may influence how an individual responds to exposure to external toxins such as carcinogens.^135^ A cohort of rural India also showed a decrease in the concentration of cyclohexanecarboxylic acid, an environmental contaminant, in the stool of centenarians compared to young people, and the authors hypothesized that the gut microbiota may be metabolizing it in the older individuals^68^ and hence mitigating any potential toxicity.

When interpreting much of these data, it is important to take into account that pathways does not always correlate with abundance of produced metabolites,^136^ indicating that there is yet more layers or complexity between the host and gut microbiome underlying how it might affect aging more broadly. Another method of examining metabolic potential is using proteomics to examine abundance of proteins present in the microbiome as opposed to gene content only. A recent proteomics-based study revealed a very high degree of correlation between age and a decreasing abundance of proteins involved in indole and tryptophan biosynthesis within the microbiome.^137^ Clearly, there is substantial work to be done to determine what functional components of the gut microbiome contribute most to healthy aging, and the mechanisms by which they exert their effect (systemic absorption of metabolites, immune interaction, local effects on gut physiology, etc.).

## Microbiome-gut brain axis

The “microbiota-gut-brain axis”, the relationship between the brain and commensal bacteria of the gut, offers a promising avenue for badly needed interventional therapies in neurodegenerative disorders. This area of study was initiated in antibiotic-treated and germ-free mice that has demonstrated a role for the gut microbiome in neurogenesis, myelination, and maintenance of blood-brain-barrier integrity.^138–140^ Many neurodegenerative disorders, including Alzheimer’s disease, one of the most devastating in this category, have been linked to differences in the composition of gut microbiota,^94,141,142^ suggestive of a role in neurodegenerative disease processes.^143,144^

Recently, the pathogenesis of Alzheimer’s disease and other cognitive disorders has been linked to microbes, either causing infections or colonizing the body, as a trigger in the development of neuroinflammation.^145–147^ This process has become a key thematic framework for Alzheimer’s disease.^148^ For example, it has been shown that blood levels of LPS correlate with the extent of observed brain amyloidosis in Alzheimer’s disease patients.^149^ In Alzheimer’s disease patients, lower serum concentrations of primary bile acids and increased microbial produced secondary bile acids levels have been shown^150^ while increases in specific secondary bile acids, such as deoxycholic acid, are strongly associated with cognitive decline.^151^ In the brain tissue of Alzheimer’s disease patients, secondary bile acids not explained by human pathways have been measured,^152^ suggesting that these abnormal secondary bile acids originate from the gut microbiome and translocate to the brain. These are just a few examples in an ever expanding and complex field of study.

## Multi-omics and medical microbiome therapeutics for aging

The association between the gut microbiome and healthy aging suggests that the microbiome may represent a modifiable risk factor for health decline in older adults. However, interventions aimed at the microbiome have had mixed results. Dietary interventions on frailty with microbiome measures as part of the intervention have been performed. The most reported is the “Nu Age” trial, a multi-center one-year intervention of community dwelling older adults treated with a dietary intervention based on the Mediterranean Diet did show an impact on microbiome features, such as increases in health associated taxa such as *F. prausnitzii*, and that these microbiome features were associated with better scores on objective measures of frailty like grip strength and neuropsychiatric scores.^128^ While encouraging, these measures primarily disease-oriented outcomes and there was no significant effect on cognition.^153^ Clearly much work is needed with dietary interventions.

Probiotics, that is, administration of specific health associated bacteria taxa, in the treatment of frailty have often shown positive changes in biomarkers and microbiome features, but often lack significant patient-oriented outcomes.^154^ Targeting the microbiome specifically with probiotics for treatment of dementia has yielded improvements in biomarkers associated with cardiovascular health but no meaningful effect on cognition.^155^ The microbiome’s ability to transform specific nutrients, or prebiotics, may also be important to the success of a dietary intervention. To specifically attempt to effect microbial metabolites, there are numerous studies of specific non-digestible carbohydrates that are the hypothesized to be substrates for the production of SCFAs and preferred energy sources for SCFA producing organisms. These often show effects on specific biomarkers or microbial taxa, but again usually not on patient-oriented outcomes.^156^

Probiotics, prebiotics, dietary interventions alone usually do not show large impacts on aging-related diseases like dementia^157^ or frailty.^158^ There are many potential reasons for this. Beneficial strains may not colonize all individuals who receive a probiotics; an intensive study of a probiotic cocktail in volunteers showed that certain individual’s microbiomes may also be “resistant” to colonization by beneficial commensals, and that the abundance of these strains decreases over time.^159^ It may be that shifts in the microbiome alone driven by diet and prebiotics may not be sufficient to drive changes in deteriorating health associated with aging. As previously mentioned, many of the mechanisms behind positive associations of microbiome features and hallmarks of healthy aging still being explored. Perhaps, it is not surprising that microbiome-based interventions have not yielded consistent positive results when we do not know what specific pathways and processes are being targeting with these interventions. With better mechanistic understanding from *in vitro* and animal model systems or interventional trials, more effective microbiome-based interventions may come in the future.

Another explanation may be that the microbiome may need to be targeted along with other interventions aimed at improving health. As the ability to collect, manage, and analyze increasingly large and complex datasets evolves, multidimensional or “multi-omics” approaches to defining biological age^160^ and aging-related phenomenon like dementia^161^ are emerging. In this context, the gut microbiome likely represents a dimension of aging process in combination with immune function, organ system health, genetics, and so on.^162^ This may be why, as previously mentioned, a single intervention targeting the microbiome may not be particularly effective, but multi-domain studies that combine interventions including diet, exercise, cardiovascular risk factor modification have shown promise^163,164^ and studies of the impact of these multi-domain interventions on the gut microbiome with be forthcoming.^165^ Decades of research examining the relationship between the gut microbiome and aging has suggested that it can impact the health of humans as they age and represents a promising target to promote healthy aging. A better mechanistic understanding of how this occurs in healthy older adults and how it fits in with the aging process will be key to making microbiome health a cornerstone of longevity and healthy aging as we suspect it will be in the future.
